# Living in the Fast Lane: Evidence for a Global Perceptual Timing Deficit in Childhood ADHD Caused by Distinct but Partially Overlapping Task-Dependent Cognitive Mechanisms

**DOI:** 10.3389/fnhum.2017.00122

**Published:** 2017-03-20

**Authors:** Ivo Marx, Steffen Weirich, Christoph Berger, Sabine C. Herpertz, Stefan Cohrs, Roland Wandschneider, Jacqueline Höppner, Frank Häßler

**Affiliations:** ^1^Department of Child and Adolescent Psychiatry, Neurology, Psychosomatics and Psychotherapy, University Medicine RostockRostock, Germany; ^2^Department of General Psychiatry, University of HeidelbergHeidelberg, Germany; ^3^Department of Psychiatry and Psychotherapy, University Medicine RostockRostock, Germany

**Keywords:** ADHD, time, timing, time perception, predictors, methylphenidate

## Abstract

Dysfunctions in perceptual timing have been reported in children with ADHD, but so far only from studies that have not used the whole set of timing paradigms available from the literature, with the diversity of findings complicating the development of a unified model of timing dysfunctions and its determinants in ADHD. Therefore, we employed a comprehensive set of paradigms (time discrimination, time estimation, time production, and time reproduction) in order to explore the perceptual timing deficit profile in our ADHD sample. Moreover, we aimed to detect predictors responsible for timing task performance deficits in children with ADHD and how the timing deficits might be positively affected by methylphenidate. Male children with ADHD and healthy control children, all aged between 8 and 13 years, participated in this longitudinal study with three experimental sessions, where children with ADHD were medicated with methylphenidate at the second session but discontinued their medication at the remaining sessions. The results of our study reveal that children with ADHD were impaired in all timing tasks, arguing for a general perceptual timing deficit in ADHD. In doing so, our predictor analyses support the notion that distinct but partially overlapping cognitive mechanisms might exist for discriminating, estimating/producing, and reproducing time intervals. In this sense, working memory deficits in terms of an abnormally fast internal counting process might be common to dysfunctions in the time estimation/time production tasks and in the time reproduction task, with attention deficits (e.g., in terms of disruptions of the counting process) additionally contributing to time estimation/time production deficits and motivational alterations additionally contributing to time reproduction deficits. Methylphenidate did not significantly alter performance of the ADHD sample, presumably due to limited statistical power of our study. The findings of our study demonstrate a pivotal role of disturbed working memory processes in perceptual timing task performance in childhood ADHD, at the same time broadening the view for additional attentional and motivational determinants of impaired task performance.

## Introduction

Time exists from the beginning of each individual's life. Starting during intrauterine life, the unborn child is exposed to external time cues like the mothers' sleep-wake-cycle, feeding times, or sounds. Following childbirth, the infant is successively confronted with increased influence from external clocks, and as a consequence thereof with increasing requirements concerning temporal self-organization. As one gets older, synchronizing activities and keeping deadlines and appointments becomes more and more important, and the extent to which the individual is able to cope with these demands decides to which extent he or she meets performance or social requirements. In addition to these cognitive-structural aspects of timing, the perception of time can also be influenced by our emotions: We wish to escape from certain situations that we perceive as boring and never-ending, but on the other hand, time is running out when we are engaged in activities that give us pleasure. In this way, time is also entangled with our individual needs and desires. Therefore, time is not only a static external framework, but is also subject to variability of individual perception.

Deficits in adaptation to externally imposed schedules or subjectively perceived time are often seen in children with attention-deficit/hyperactivity disorder (ADHD), and a significant number of the diagnostic criteria of the disorder reminds of behavioral manifestations of timing deficits (American Psychiatric Association, 2013). Therefore, some researchers understand ADHD as a timing disorder (Barkley, 1997). They argue that a disturbed sense of time results from an impaired ability to withhold immediate behaviors, which makes it impossible for the individual to bring his or her behavior under the control of higher-order and goal-directed cognitive processes, the so-called executive functions (EF). One of these functions is working memory (WM) which denotes the ability to hold information in mind while pursuing future goals, thereby adding a temporal dimension to human cognition by giving rise to hindsight and forethought (Barkley, 1997). As a result of impaired WM, “ADHD is associated with a form of temporal myopia or time blindness concerning the direction of behavior toward conjectured future events. The behavior of those with ADHD is more controlled by the temporal now than by internally represented information pertaining to past and future” (Barkley, 1997, p. 275). In short, Barkley (1997) proposes that inhibitory deficits prevent EFs—amongst them WM—from working properly, such that temporal information cannot be sufficiently coded within the WM. As a result, timing dysfunctions emerge in subjects with ADHD which manifest themselves at the behavioral level in the form of impulsivity. Likewise, Rubia et al. (2009) argues that abnormalities in timing functions, i.e., deficits in motor timing, perceptual timing, and temporal foresight, are elementary to impulsiveness, at the same time giving a more central role to timing deficits in the etiology of impulsive behaviors in subjects with ADHD. Both models effectively correspond insofar as they stress a central role of WM in the development of timing deficits, and the association between WM and time processing yields support from meta-analytical research: The biological substrate of WM has been identified within a fronto-parietal network (Wager and Smith, 2003; Owen et al., 2005; Rottschy et al., 2012) which is also involved in timing tasks (Wiener et al., 2010). Additionally, WM deficits were found to predict timing dysfunctions in children and adolescents with ADHD (McInerney and Kerns, 2003; Bauermeister et al., 2005).

In the domain of ADHD, a variety of perceptual timing paradigms have been applied, including tasks that require temporal judgments in the range of milliseconds and those in the range of seconds. The former evaluate the ability of the child to discriminate between stimuli that differ in their presentation time for only several milliseconds (time discrimination tasks), imposing requirements especially on perceptual acuity. The latter, in contrast, require additional cognitive resources, as they ask the child to provide a verbal duration estimation of a previously presented stimulus (time estimation), to produce a previously specified time interval, e.g., by pressing a response button (time production), or to infer the duration of a stimulus that had been previously presented for a defined time interval and to indicate this time interval subsequently by pressing a response button (time reproduction; see Grondin, 2010, for an overview). These four paradigms (i.e., time discrimination, time estimation, time production, and time reproduction) have been subsumed under the umbrella term “perceptual timing,” as they require the estimation of explicitly attended time intervals (Noreika et al., 2013). Research over the past 15 years has shown that perceptual timing is impaired in children and adolescents with ADHD, which is especially true for their time discrimination and time reproduction abilities.

In children and adolescents with ADHD, time discrimination deficits have been objectified both in the auditory (Toplak et al., 2003; Toplak and Tannock, 2005; Himpel et al., 2009) and in the visual domain (Smith et al., 2002; Rubia et al., 2007; Yang et al., 2007; Vloet et al., 2010), using different baselines for the duration of the comparison stimulus as the initial starting point (auditory: between 50 and 1,000 ms; visual: between 300 and 1,200 ms). Thus, time discrimination deficits seem to exist independent from perceptional modality (Toplak and Tannock, 2005), but there is some evidence that performance deteriorates with increasing baseline length (Yang et al., 2007) and increasing duration difference between the comparison stimuli (Valko et al., 2010; but see also Smith et al., 2008, for a negative finding).

Verbal time estimation seems rather unimpaired in children and adolescents with ADHD (Barkley et al., 2001; Smith et al., 2002; Meaux and Chelonis, 2003; Bauermeister et al., 2005), although one single study reported an overestimation of time intervals in the range of several seconds (Hurks and Hendriksen, 2011). The body of literature is more heterogeneous with regard to time production performance in children and adolescents with ADHD. Three studies tested the production of a 1 s interval. Whereas two of these studies found children with ADHD to underproduce this time interval (Rommelse et al., 2008; Luman et al., 2009), one study did not, but children with ADHD were found to generate a numerically higher number of extreme underproductions and a larger response variability (Van Meel et al., 2005). Only two studies so far have examined time production performance in the range of several seconds. One of these studies found larger absolute discrepancy scores, i.e., larger deviations from the time intervals that have to be produced, as well as an underproduction of these time intervals, i.e., a lower ratio between the manually produced and the visually presented time intervals, in children with ADHD (Huang et al., 2012). Marx et al. (2010) also found lower accuracy scores in the ADHD group, but an in-depth analysis of this finding shows that subjects with ADHD underproduced the time intervals, whereas controls overproduced the time intervals, with the absolute deviation from “perfect performance” being equal between both groups. Therefore, it seems questionable if the numerically lower values in the ADHD group actually represented a timing deficit in their study. In conclusion, the body of literature seems to indicate that time production deficits might exist in children and adolescents with ADHD, although they are not yet sufficiently examined, at least in the range of several seconds.

The largest body of literature exists for time reproduction tasks, and these studies demonstrate larger absolute discrepancy scores, i.e., larger deviations from the time intervals that have to be reproduced, in children and adolescents with ADHD (Barkley et al., 2001; Kerns et al., 2001; McInerney and Kerns, 2003; Bauermeister et al., 2005; Valko et al., 2010), with a steeper increase of this error with increasing interval length when compared with controls (Kerns et al., 2001; Hwang et al., 2010; but see also McInerney and Kerns, 2003), and independent from the presentation mode (Rommelse et al., 2007; Plummer and Humphrey, 2009). Two studies, in contrast, found no increased absolute discrepancy scores at short interval durations, where the involvement of executive functions might be minimized (Smith et al., 2002; Toplak et al., 2003). With regard to the direction of the time reproduction error, studies are more inconsistent, as they found subjects with ADHD to reproduce shorter time intervals, averaged across different interval lengths, when compared with controls (Kerns et al., 2001), to display a steeper decline of reproduction times with increasing interval length (Hurks and Hendriksen, 2011), to overestimate short time intervals and to underestimate long time intervals (McInerney and Kerns, 2003), or even to perform equally to controls (Marx et al., 2010).

Barkley (1997) postulates WM dysfunctions to interfere with timing deficits in children and adolescents with ADHD, and it has indeed been found that WM deficits which have been identified in this sample (Martinussen et al., 2005) are predictive for time reproduction deficits in ADHD (McInerney and Kerns, 2003; Bauermeister et al., 2005). However, children and adolescents with ADHD are impaired in both time reproduction and time discrimination tasks, and a recent meta-analysis identified partially overlapping but distinct networks for the processing of short (in the range of milliseconds) and long (in the range of seconds) temporal intervals (Wiener et al., 2010), indicative for differential brain activations during the processing of time discrimination and time reproduction tasks. More precisely, a “core” timing network including the right inferior frontal gyrus (IFG) and the bilateral supplementary motor area (SMA) has been identified, contributing to timing across different modalities and durations (Wiener et al., 2010). Beyond this, tasks that operate in the millisecond range such as time discrimination tasks specifically involve striatal (i.e., caudate, putamen) and cerebellar regions, whereas tasks that operate in the range of several seconds such as interval production tasks specifically involve the cingulate cortex. Furthermore, these two networks substantially overlap with regard to further regions of the frontal (i.e., superior and middle frontal; precentral) and parietal (i.e., inferior parietal) cortex (Wiener et al., 2010), and it has been suggested that fronto-parietal involvement is especially important for timing tasks involving several seconds, as attention and WM requirements increase with interval length (see Noreika et al., 2013). Thus, WM might be rather less important for successful time discrimination when compared with time reproduction, but available data are inconsistent:

Only two studies so far have examined the association between WM and time discrimination in adolescent ADHD samples while simultaneously controlling for further potential predictors (Toplak et al., 2003; Toplak and Tannock, 2005). In both studies, WM performance predicted time discrimination deficits; whereas one study found this association only to be true for long baseline stimulus durations (1,000 ms) but not for short stimulus durations (200 ms) (Toplak and Tannock, 2005), the other study found an association at short stimulus durations (400 ms) as well (Toplak et al., 2003). All further analyses presented in the literature were correlational by nature, and these draw the same inconsistent picture: two studies failed to find an association between WM and time discrimination performance at short stimulus durations (Yang et al., 2007; Vloet et al., 2010) but found an association at long stimulus durations (Yang et al., 2007), and one further study failed to find an association between both measures at long stimulus durations (Smith et al., 2002) in children and adolescents with ADHD. Importantly, the available literature is not only inconclusive with regard to the association between WM and time discrimination performance, but also concerning the association between WM and time reproduction performance in the range of seconds, reporting only one predictive association (McInerney and Kerns, 2003) and one positive correlation (Bauermeister et al., 2005), but also two negative findings (Kerns et al., 2001; Toplak et al., 2003), such that there is still need for a clarification for both kinds of timing tasks.

It is important to note that beyond WM, a more diversified set of cognitive functions promotes performance in perceptual timing tasks. Whereas WM is required for holding temporal reference information online (e.g., the interval length to be reproduced in a time reproduction task), attention to time (e.g., attention allocation to the ongoing task; adjustment of motor responses with regard to the defined time intervals) and inhibition of premature responding seem especially important (Rubia et al., 2009). In ADHD, deficits in these cognitive domains interfere with timing performance, causing timing dysfunctions in the affected subjects (Noreika et al., 2013). With regard to further potential predictors of impaired timing task performance, researchers have indeed identified a more complex set of executive dysfunctions beyond WM (i.e., interference control, inhibition, and WM) as well as clinical inattentive symptoms to predict larger time reproduction discrepancy scores in children with ADHD (McInerney and Kerns, 2003; Bauermeister et al., 2005; Hurks and Hendriksen, 2011). Likewise, neuropsychological measures of inattention and inhibition, as well as hyperactivity ratings, were correlated with time discrimination, at least at long stimulus durations (Rubia et al., 2007). However, those studies implementing regression analyses (Toplak et al., 2003; Toplak and Tannock, 2005) consistently failed to demonstrate that parental and teacher ratings of ADHD symptoms predict time discrimination deficits beyond neuropsychological measures, irrespective of stimulus duration. These findings not only suggest that further neuropsychological domains beyond WM contribute to timing dysfunctions in children and adolescents with ADHD, but they also raise the question if neuropsychological measures, when compared with behavioral ADHD symptom ratings, might be the “better” predictors of timing dysfunctions in this population.

In recent years, meta-analytical evidence has been accumulating that methylphenidate (MPH) increases frontal-striatal brain activation in children and adolescents with ADHD (Rubia et al., 2014), at the same time improving cognitive functioning (Coghill et al., 2014), school-related on-task behavior (Prasad et al., 2013), and behavioral symptom ratings (Punja et al., 2013). In the domain of timing, two studies found MPH to reduce time discrimination deficits in children and adolescents with ADHD (Smith et al., 2013; Rubia et al., 2014), whereas two studies did not (Rubia et al., 2003, 2009). Furthermore, one study did not find MPH to reduce time reproduction deficits in children and adolescents with ADHD (Barkley et al., 1997).

The aim of this study was to bring together the whole set of perceptual timing paradigms which, up to now, have been applied to investigating timing in ADHD within one study design in order to rule out differences in task parameters (e.g., interval lengths, sensory domains) and sample composition which might have produced heterogeneity in the results between previous studies. This holistic approach allows an analysis of associations between all four perceptual timing tasks within one single study and the identification of shared or distinct cognitive mechanisms that affect task performance. Furthermore, we considered the question whether the same set of predictors (neuropsychological measures; parental ADHD symptom ratings) helps to explain both time reproduction and time discrimination dysfunctions in children with ADHD, and if the ADHD symptom ratings predict timing dysfunctions beyond the neuropsychological measures. Based on the literature reviewed above, we expected children with ADHD to be impaired in the time discrimination and the time reproduction task, but not in the time estimation and the time production task. Furthermore, we hypothesized that MPH improves timing performance in children with ADHD, at least in the domain of time discrimination. In addition, we expected WM to predict timing deficits in ADHD, with a stronger linkage with time reproduction when compared with time discrimination. Lastly, we assumed measures of inattention and inhibition to predict timing dysfunctions as well, with a larger predictive power for neuropsychological measures when compared with observational measures in terms of behavioral symptom ratings.

This is the first longitudinal study using three experimental sessions in order to assess timing deficits and medication effects in children with ADHD, simultaneously taking effects of practice into account. In this sense, timing deficits in the ADHD group are rigorously defined as inferior timing task performance as measured across all three sessions (main effect of group membership), and the possible performance improving effect of methylphenidate was rigorously defined as a superior performance at the second session where the children with ADHD were taking their prescribed MPH medication when compared with both initial (first session) and subsequent (third session) task performance when the children with ADHD discontinued their medication (interaction effect between group membership and number of session).

## Materials and methods

### Participants

We examined male children with ADHD who were aged 8–13 years, as well as typically developing control children. The patients were recruited from the inpatient and outpatient clinics of the Department of Child and Adolescent Psychiatry, University Medicine Rostock, Germany. Control subjects were recruited from primary and secondary schools via flyers that were distributed by their teachers after a consultation with the headmasters.

For all children, the diagnostic procedure included the German version of the Kiddie-Sads-Present and Lifetime Version (K-SADS-PL, Kaufman et al., 1997), which is a semi-structured interview to assess lifetime and current psychiatric diagnoses based on DSM-IV criteria. The diagnosis of ADHD was assessed by an experienced senior child and adolescent psychiatrist (S. W.). For a diagnosis of ADHD, children had to currently fulfill the relevant number of diagnostic criteria, including those related to age of onset. To obtain parental ratings of the children's behavioral problems, the German version of the Child Behavior Checklist (CBCL; Döpfner and Lehmkuhl, 1998) was used. The severity of ADHD symptoms, according to the DSM-IV subtype classification, was assessed by means of the German Parental and Teacher Report on ADHD symptoms (FBB-HKS; Döpfner and Lehmkuhl, 1998). ODD and CD symptoms were recorded by means of the German Parental and Teacher Report on disruptive behavior symptoms (FBB-SSV; Döpfner and Lehmkuhl, 1998), using a four-point rating scale (0 = not at all; 3 = very much). The children's IQ was assessed by means of a German adaptation of Cattell's Culture Fair Intelligence Tests that included two age-related subscales (Weiss and Osterland, 1997; Weiss, 1998). Exclusion criteria for all participants included an IQ below 80, neurological or endocrine disorders known to affect brain function, previous head injury, current depressive disorder, and lifetime schizophrenia spectrum disorder.

The initial sample consisted of 22 children with ADHD and 21 controls. As four children with ADHD and three control children discontinued before completing all three experimental sessions and one child with ADHD was newly diagnosed with epilepsy and was therefore excluded, the final sample included 17 boys with ADHD and 18 male controls. In the ADHD group, seven subjects were primarily inattentive, three were primarily hyperactive/impulsive, and seven subjects suffered from the combined subtype. Four of them were inpatients, and 13 were outpatients. Within the ADHD sample, one child suffered from reactive attachment disorder, two children suffered from a specific reading disorder, and two children suffered from non-organic enuresis and encopresis, respectively. No psychiatric disorders were found within the control sample. Two children with ADHD were initially drug-naïve but were planned to be medicated with MPH during their inpatient treatment, while the others where pre-medicated with MPH. Eight children were medicated with immediate-release MPH (Medikinet: *n* = 3; Equasym: *n* = 4; Ritalin: *n* = 1; mean dosage: 25.63 ± 6.23 mg, range 15–30 mg; mean body weight 36.24 ± 10.16 kg), and nine children were medicated with delayed-release MPH (Medikinet Retard: *n* = 8; Ritalin LA: *n* = 1; mean dosage: 30.00 ± 8.66 mg, range 20–40 mg; mean body weight 41.09 ± 11.02 kg). The relative MPH dosage was about 0.7 mg/kg.

ADHD subjects and controls did not differ in terms of IQ, but there were subtle age differences between the groups. Group differences were also found for the FBB-HKS and FBB-SSV subscales as well as for most of the CBCL subscales. As one parent did not return the questionnaires, clinical data are available for only 16 out of 17 children with ADHD. Sample characteristics are presented in Table 1.

**Table 1:** **Sample characteristics**.

	**ADHD (*N* = 16) *M* (*SD*)**	**CON (*N* = 18) *M* (*SD*)**	***t*_(33)_; *F*_(1, 31)_**
Age	10.63 (1.58)	9.45 (1.46)	2.30^*^
IQ	97.69 (10.75)	104.67 (14.17)	1.60
**FBB-HKS**			
Inattention	7.13 (1.20)	4.17 (1.43)	66.38^***^
Hyperactivity	7.38 (1.41)	4.72 (0.83)	61.68^***^
Impulsiveness	7.25 (1.88)	4.78 (1.00)	34.85^***^
Sum Score	7.25 (1.39)	3.94 (1.39)	69.74^***^
**FBB-SSV**			
Oppositional-Aggressive	7.06 (1.84)	3.89 (1.61)	46.66^***^
Dissocial-Aggressive	6.56 (2.03)	4.56 (0.86)	32.91^***^
Sum Score	6.81 (1.97)	3.83 (1.25)	46.18^***^
**CBCL**			
Withdrawn	59.00 (7.31)	54.44 (5.79)	5.00
Somatic Complaints	57.38 (8.24)	52.78 (5.09)	2.29
Anxious/Depressed	59.00 (9.01)	52.56 (4.78)	8.44^**^
Social Problems	57.56 (9.17)	50.61 (1.34)	9.05^**^
Thought Problems	56.63 (7.51)	51.17 (2.68)	6.70^*^
Attention Problems	64.25 (8.08)	51.17 (2.33)	60.14^***^
Delinquent Behavior	62.06 (12.52)	51.89 (4.46)	19.13^***^
Aggressive Behavior	61.38 (10.39)	50.83 (2.09)	31.47^***^
Internalizing Problems	58.81 (10.71)	49.22 (8.52)	10.10^**^
Externalizing Problems	60.06 (13.48)	43.61 (7.80)	40.14^***^

*ADHD, ADHD group; CON, control group. N, number of subjects; M, median; SD, standard deviation. FBB-HKS, parental report on ADHD symptoms according to the DSM-IV criteria; FBB-SSV, parental report on CD/ODD symptoms according to the DSM-IV criteria; CBCL, Child Behavior Checklist. t, t-value; F, F-value*.

* p < 0.05;

** p < 0.01;

*** *p < 0.001*.

### Procedure

Prior to study participation, all children and their parents were informed about the aim of the study unspecifically, i.e., they were told that they will take part in a computer-based examination of their time perception abilities as well as their attention and memory processes in a longitudinal study, comprising three experimental sessions. All sessions took place between 8 and 12. In order to minimize effects of practice, we chose a relatively large time lag between the three sessions. In doing so, about 5–6 weeks were lying in-between the first and the second session [ADHD: 38.59 ± 12.96 days; CON: 37.89 ± 11.90 days; *t*_(33)_ = −0.17, *p* = 0.87, *ns*], and about 7–11 weeks were lying in-between the second and the third session [ADHD: 77.47 ± 57.21 days; CON: 52.89 ± 22.54 days; *t*_(33)_ = −1.69, *p* = 0.11, *ns*]. At sessions one and three, children with ADHD discontinued their MPH medication at least 24 h (immediate-release) or 48 h (delayed-release) before testing, whereas they were medicated at session two. The second session started in relation to MPH intake and was guided to be around the peak of MPH plasma concentrations with about 1.5–2.5 h for immediate-release MPH formulations and between 2.5 and 5.5 h for delayed-release formulations (Patrick and Markowitz, 1997; Kimko et al., 1999; Challman and Lipsky, 2000; Coghill et al., 2013). The study was conducted in a quiet room, and only the experimenter and the child were present. Before performing the tasks, the children were given comprehensive instructions and were asked to repeat the instructions using their own words. Then, the children were asked to work in a quiet and concentrated way. The total duration of neuropsychological testing was ~1 h. At the first session, the parents filled in the questionnaires while the children worked on the tasks.

The study was conducted in accordance with the latest version of the Declaration of Helsinki. The ethics committee of the Faculty of Medicine of the University of Rostock specifically approved this study. All parents and all children gave their informed written consent. The families were compensated for their time and expense with 10 Euros per session.

### Tasks and measures

#### Time discrimination task

Participants were shown a red and a green circle in quick succession, which hardly differed in the duration of their presentation. They were then required to decide which of the circles was presented for a longer duration (Smith et al., 2002). One of the circles was always presented with a duration of 1,000 ms; the other one was initially presented with a duration of 1,300 ms, but was successively shortened by 15-ms intervals. In each successive trial, colors, and positions were randomly interchanged in order to rule out guessing strategies. The two circles were separated by a fixation cross, which was shown for 800 ms. Subsequent to the presentation of the second circle, a delay of 500 ms was introduced, followed by the instruction to choose one of the circles by responding with either the left or the right mouse button. Correct answers were followed by a reduction in the presentation duration of the longer circle by 15 ms, while incorrect answers were followed by an increase of 15 ms. This staircase method was introduced by Levitt (1971). The point of subjective equality between the circles, i.e., the point at which subjects failed to discriminate the presentation duration of the circles adequately and assessed them as being equal, served as a dependent variable. The sensitivity threshold was computed according to Smith et al. (2002).

#### Time production, time estimation, and time reproduction task

Timing in the range of several seconds was assessed by a time production and a time reproduction paradigm (Meaux and Chelonis, 2003). In the time production task, subjects saw a number on the screen, which was a time in seconds, and were asked to press the left mouse button until they were under the impression that this time span had elapsed. In the time reproduction task, yellow “smiley faces” were presented for a certain time interval. Participants then had to infer the duration for which the smileys were shown on the screen (time estimation) and, again, were asked to press the left mouse button as long as the “smiley” had occurred earlier on (time reproduction). During the button press phase, a green smiley was displayed on the screen. In all the timing tasks, subjects were explicitly instructed to count the seconds in their heads. The time intervals were 2, 6, 12, 24, 36, and 48 s. In both tasks, the time intervals were presented twice, in two successive blocks, and were randomized within the blocks. In the time reproduction task, the presentation of the smiley was signaled by a 3-s countdown. To rule out the possibility that overestimations and underestimations would average each other out, the absolute value of the deviation between the specified and the produced time interval, as a measure of accuracy, served as a dependent variable, reflecting the overall magnitude of error regardless of its direction. Additionally, an accuracy coefficient score was computed, whereby the produced time interval was divided by the specified time interval, reflecting under- (scores lower than 1.00) and over-reproductions (scores higher than 1.00).

#### Working memory

WM was assessed by means of the digit span subtest of a German adaptation of the Wechsler Memory Scale–Revised (Härting et al., 2000). The children were presented with sequences of digits of increasing length and were then asked to repeat these sequences immediately either forwards (digit span) or in the reverse order (digit span backwards). The task was terminated when the child was not able to reproduce two sequences of the same length correctly. Whereas the digit span subtest assesses the passive short-term storage of information, the digit span backwards subtest assesses both maintenance and manipulation of information (central executive) according to Baddeley (1992) conceptualization of WM. The number of correctly reproduced digits in each subtest served as a dependent variable.

#### Continuous performance task (CPT)

In this task, 525 letters were randomly presented on the screen, and the children had to press the left mouse button as fast as possible if the letter “X” appears subsequent to the letter “A.” The task comprised 52 target-sequences (“A–X”) and 20 “A” without “X” to measure omission errors. Each letter was presented for 250 ms, and the inter-stimulus interval was 1,500 ms. The task was preceded by a practice trial that contained 30 letters and four targets. The number of omission errors, false positives (FP), median response time for correctly identified targets (MDRT), and response time variability for correctly identified targets (SDRT) served as dependent variables.

### Statistical analyses

The data were analyzed using SPSS version 17 (SPSS Inc., Chicago, IL, USA). Tests of the group differences in sociodemographic data were performed using *t*-tests for independent samples, and the group differences in clinical data were assessed by means of univariate analyses of variance (ANOVAs). As age significantly differed between the two groups, age was entered as a covariate in these analyses. The group differences in the dependent variables were analyzed using repeated-measurements ANCOVAs with the diagnostic group (subjects with ADHD; controls) as the between-subjects factor and the three experimental sessions as the within-subject factor, and age as the covariate. Due to non-singular cell covariance matrices in the primary analyses, data from the time production, the time estimation, and the time reproduction task were aggregated into short (2 and 6 ms), intermediate (12 and 24 ms), and long (36 and 48 ms) time intervals. The assumption of sphericity was tested using the Mauchly-Test, and when sphericity was violated, Greenhouse-Geisser correction was implemented. In cases of significant main or interaction effects, *post-hoc* comparisons (*post-hoc* pairwise comparisons which were corrected using the Bonferroni procedure; paired-samples *t*-tests; univariate ANCOVAs) were conducted. Prior to the analyses, the raw data were z-transformed to examine extreme outliers (*z* > 3.0), and these outliers were replaced by the respective group means. The significance level for all of the tests was *p* ≤ 0.05. The partial eta-squared (η_*p*_^2^) is reported as a measure of the effect size. Due to excessive demand, one subject with ADHD discontinued the CPT, leaving data from 16 subjects with ADHD for this task.

## Results

For all paradigms, only significant between- and within-subject main effects and interaction effects (*p* < 0.05) are reported. The means and standard deviations for all dependent measures can be derived from Table 2.

**Table 2:** **Measures of task performance**.

	***t1***		***t2***		***t3***				
	**ADHD**	**CON**	**ADHD**	**CON**	**ADHD**	**CON**	**Within**	**Between**	**Within × between**
	***M (*SD*)***	***M (*SD*)***	***M (*SD*)***	***M (*SD*)***	***M (*SD*)***	***M (*SD*)***		***F***	
**TIME DISCRIMINATION**									
Sensitivity threshold	231.18 (68.10)	217.78 (56.02)	236.03 (34.10)	215.42 (56.68)	235.15 (40.38)	196.53 (67.21)		9.36^**^	
**TIME ESTIMATION**									
Absolute error	5833.33 (3556.83)	3574.07 (2610.29)	4946.08 (4020.77)	3041.12 (1891.27)	4186.27 (1858.38)	3112.75 (1772.37)	5.40^*^^a^	13.06^***^	
Accuracy score	1.15 (0.32)	1.14 (0.18)	1.16 (0.30)	1.06 (0.16)	1.18 (0.22)	1.05 (0.16)	4.61^*^^a^	3.79^*^	7.53^**^^c^
**TIME PRODUCTION**									
Absolute error	6339.12 (1976.85)	3831.54 (2091.49)	7720.99 (4201.73)	4834.50 (2719.33)	8233.64 (3083.67)	6548.13 (4022.01)	11.27^***^^a^	12.94^***^	10.62^***^^c^
Accuracy score	0.80 (0.21)	0.88 (0.16)	0.78 (0.29)	0.81 (0.17)	0.66 (0.22)	0.87 (0.29)		7.69^**^	
**TIME REPRODUCTION**									
Absolute error	5338.91 (3468.22)	3083.89 (1118.56)	4873.23 (3324.10)	3177.64 (1543.74)	5934.71 (3357.17)	3934.67 (2437.18)	8.03^**^^a^	12.10^***^	11.03^***^^c^
Accuracy score	0.89 (0.19)	0.92 (0.09)	0.84 (0.16)	0.93 (0.12)	0.79 (0.14)	0.90 (0.13)	3.60^*^^b^	6.12^*^	3.88^*^^c^3.51^*^^d^
**WORKING MEMORY**									
Digit span	5.24 (1.25)	5.28 (1.02)	5.29 (0.85)	5.44 (1.04)	5.18 (1.13)	5.72 (0.90)		5.82^*^	
Digit span backwards	3.76 (1.09)	3.89 (0.83)	3.88 (1.05)	4.33 (1.03)	3.92 (0.24)	4.39 (1.04)		5.97^*^	
**CPT**									
Omission errors	2.63 (2.47)	1.06 (1.21)	0.56 (0.63)	1.50 (1.89)	0.94 (2.05)	1.00 (1.24)			9.39^***^^d^
Commission errors	3.13 (2.78)	3.17 (2.46)	1.75 (1.39)	2.39 (2.59)	2.13 (3.12)	1.89 (1.84)			
MDRT	331.92 (68.41)	348.39 (111.86)	332.23 (73.27)	339.21 (52.36)	342.93 (53.99)	339.21 (52.36)			
SDRT	111.03 (46.39)	102.14 (35.21)	91.46 (34.81)	113.75 (35.23)	122.17 (47.51)	111.12 (36.15)			3.52^*^^d^

t1, first session without MPH; t2, second session on MPH; t3, third session without MPH; ADHD, ADHD group; CON, control group. Within, main effect of the within-subject factor (

a *time interval*,

b experimental session); Between, main effect of the between-subject factor (diagnostic group); Within × Between, interaction effect of the within-subject factor and the between-subject factor (

c time interval × diagnostic group;

d *session × diagnostic group). M, median; SD, standard deviation. Sensitivity Threshold, the point at which two stimuli that are presented in the range of milliseconds and that differ in length are perceived as being equal (Time Discrimination); Absolute Error, the absolute value of the deviation between specified and produced time interval in milliseconds (Time Estimation, Time Production, Time Reproduction); Accuracy Score, the estimated time interval divided by the time interval to be produced in order to evaluate under- or overestimation in milliseconds (Time Estimation, Time Production, Time Reproduction); Digit Span, number of digits repeated; Digit Span Backwards, number of digits repeated backwards (Working Memory); Hits, number of correctly identified targets (“A–X”); Omission Errors, number of targets that were not identified; Commission Errors, non-targets that were identified as targets “A-not-X”); MDRT, median reaction time in milliseconds; SDRT, standard deviation of reaction time for correctly identified targets in milliseconds (CPT). F, F-value*.

* p < 0.05;

** p < 0.01;

*** *p < 0.001*.

### Time discrimination

Children with ADHD displayed a lower sensitivity threshold when compared with controls, *F*_(1, 32)_ = 9.36, *p* = 0.004, η_*p*_^2^ = 0.23.

### Time estimation

#### Absolute error

All children, irrespective of their group membership, displayed larger absolute estimation errors with increasing length of the time intervals, *F*_(2, 64)_ = 5.40, *p* = 0.02, η_*p*_^2^ = 0.14 (short vs. intermediate: *p* < 0.001; intermediate vs. long: *p* < 0.001; short vs. long: *p* < 0.001). Children with ADHD displayed a larger overall absolute estimation error when compared with controls, *F*_(1, 32)_ = 13.06, *p* = 0.001, η_*p*_^2^ = 0.29.

#### Performance accuracy

All children performed better when the time interval length increased, i.e., their overestimation decreased, *F*_(2, 64)_ = 4.61, *p* = 0.02, η_*p*_^2^ = 0.13 (short vs. intermediate: *p* < 0.001; intermediate vs. long: *p* = 0.001; short vs. long: *p* < 0.001). This effect developed differentially in both groups, *F*_(2, 64)_ = 7.53, *p* = 0.003, η_*p*_^2^ = 0.19, as the ADHD group displayed a steeper performance increment [controls: short vs. intermediate: *t*_(17)_ = 3.61, *p* = 0.002; intermediate vs. long: *t*_(17)_ = 1.21, *p* = 0.24, *ns*; short vs. long: *t*_(17)_ = 4.53, *p* < 0.001; ADHD: short vs. intermediate: *t*_(16)_ = 4.24, *p* = 0.001; intermediate vs. long: *t*_(16)_ = 4.97, *p* < 0.001; short vs. long: *t*_(16)_ = 5.17, *p* < 0.001]. As a result, children with ADHD overestimated the short, *F*_(1, 32)_ = 7.44, *p* = 0.01, η_*p*_^2^ = 0.19, and the intermediate, *F*_(1, 32)_ = 4.12, *p* = 0.05, η_*p*_^2^ = 0.11, but not the long time intervals, *F*_(1, 32)_ = 0.27, *p* = 0.61, *ns*. However, across all time intervals, children with ADHD tended to overestimate the time intervals, *F*_(1, 32)_ = 3.79, *p* = 0.06, η_*p*_^2^ = 0.11.

### Time production

#### Absolute error

All children displayed larger absolute production errors with increasing length of the time intervals, *F*_(2, 64)_ = 11.27, *p* = 0.001, η_*p*_^2^ = 0.26 (short vs. intermediate: *p* < 0.001; intermediate vs. long: *p* < 0.001; short vs. long: *p* < 0.001). The absolute production error increased differentially in both groups, with a steeper increase in the ADHD group, *F*_(2, 64)_ = 10.62, *p* = 0.001, η_*p*_^2^ = 0.25. As a result, children in both groups displayed a similar performance at short time intervals, *F*_(1, 32)_ = 1.71, *p* = 0.20, *ns*, but children with ADHD showed an increased absolute production error at intermediate, *F*_(1, 32)_ = 8.91, *p* = 0.005, η_*p*_^2^ = 0.22, and at long, *F*_(1, 32)_ = 12.80, *p* = 0.001, η_*p*_^2^ = 0.29, time intervals. As a result, children with ADHD displayed a larger overall absolute production error when compared with controls, *F*_(1, 32)_ = 12.94, *p* = 0.001, η_*p*_^2^ = 0.29.

#### Performance accuracy

With regard to error direction, children with ADHD underproduced the time intervals, *F*_(1, 32)_ = 7.69, *p* = 0.009, η_*p*_^2^ = 0.19.

### Time reproduction

#### Absolute error

All children displayed larger absolute reproduction errors with increasing length of the time intervals, *F*_(2, 64)_ = 8.03, *p* = 0.006, η_*p*_^2^ = 0.20 (short vs. intermediate: *p* < 0.001; intermediate vs. long: *p* < 0.001; short vs. long: *p* < 0.001). The absolute reproduction error increased differentially in both groups, again with a steeper increase in the ADHD group, *F*_(2, 64)_ = 11.03, *p* = 0.001, η_*p*_^2^ = 0.26. It became evident that children with ADHD displayed a higher absolute reproduction error at long, *F*_(1, 32)_ = 12.52, *p* = 0.001, η_*p*_^2^ = 0.28, and at short, *F*_(1, 32)_ = 5.13, *p* = 0.03, η_*p*_^2^ = 0.14, and, as a trend, at intermediate time intervals, *F*_(1, 32)_ = 3.78, *p* = 0.06, η_*p*_^2^ = 0.11, in comparison to controls. As a result, children with ADHD displayed a larger overall absolute reproduction error when compared with controls, *F*_(1, 32)_ = 12.10, *p* = 0.001, η_*p*_^2^ = 0.27.

#### Performance accuracy

With regard to error direction, children with ADHD underreproduced the time intervals, *F*_(1, 32)_ = 6.12, *p* = 0.02, η_*p*_^2^ = 0.16. The reproduction error developed differentially in both groups with increasing length of the time intervals, *F*_(2, 64)_ = 3.88, *p* = 0.04, η_*p*_^2^ = 0.11. Whereas, controls displayed a stable performance across all three interval lengths [short vs. intermediate: *t*_(17)_ = −1.29, *p* = 0.22, *ns*; intermediate vs. long: *t*_(17)_ = 1.44, *p* = 0.17, *ns*; short vs. long: *t*_(17)_ = −0.51, *p* = 0.62, *ns*], subjects with ADHD deteriorated in their performance at long time intervals when compared with short and intermediate time intervals [short vs. intermediate: *t*_(16)_ = 0.68, *p* = 0.51, *ns*; intermediate vs. long: *t*_(16)_ = 2.76, *p* = 0.01; short vs. long: *t*_(16)_ = 2.07, *p* = 0.05]. As a consequence, children with ADHD underreproduced the intermediate, *F*_(1, 32)_ = 7.02, *p* = 0.01, η_*p*_^2^ = 0.18, and the long time intervals, *F*_(1, 32)_ = 11.34, *p* = 0.002, η_*p*_^2^ = 0.26, but not the short time intervals, *F*_(1, 32)_ = 0.22, *p* = 0.64, *ns*, in comparison to controls.

### Working memory

Controls outperformed children with ADHD with regard to both the digit span, *F*_(1, 32)_ = 5.82, *p* = 0.02, η_*p*_^2^ = 0.15, and the digit span backwards, *F*_(1, 32)_ = 5.97, *p* = 0.02, η_*p*_^2^ = 0.16.

### CPT

With the exception of longitudinal effects of repeated task exposure which are presented below, no significant effects emerged.

### Effects of repeated task exposure

In the time reproduction task, performance accuracy decreased, i.e., underreproduction increased, in all children, depending on session number, *F*_(2, 64)_ = 3.60, *p* = 0.03, η_*p*_^2^ = 0.10, with a trend for a loss of accuracy especially at the third session (first vs. second: *p* = 1.00, *ns*; second vs. third: *p* = 0.42, *ns*; first vs. third: *p* = 0.06). The underreproduction developed differentially in both groups, *F*_(2, 64)_ = 3.51, *p* = 0.04, η_*p*_^2^ = 0.10. Whereas controls displayed a stable performance across all three sessions [t1 vs. t2: *t*_(17)_ = −0.34, *p* = 0.74, *ns*; t2 vs. t3: *t*_(17)_ = 0.85, *p* = 0.41, *ns*; t1 vs. t3: *t*_(17)_ = 0.46, *p* = 0.65, *ns*], children with ADHD deteriorated in the course of time and displayed under reproduction during the third session [t1 vs. t2: *t*_(16)_ = 1.44, *p* = 0.17, *ns*; t2 vs. t3: *t*_(16)_ = 1.19, *p* = 0.25, *ns*; t1 vs. t3: *t*_(16)_ = 2.44, *p* = 0.03]. As a consequence, children with ADHD did not differ from controls in reproduction accuracy at the first session, *F*_(1, 32)_ = 0.31, *p* = 0.58, *ns*, but they slightly did at the second session, *F*_(1, 32)_ = 4.75, *p* = 0.04, η_*p*_^2^ = 0.13, and marked differences emerged at the third session, *F*_(1, 32)_ = 14.00, *p* = 0.001, η_*p*_^2^ = 0.30.

In the CPT, differential effects of repeated task exposure emerged, as both groups differed with regard to the number of omission errors, *F*_(2, 62)_ = 9.39, *p* < 0.001, η_*p*_^2^ = 0.23, and response time variability, *F*_(2, 62)_ = 3.52, *p* = 0.04, η_*p*_^2^ = 0.10, depending on session number: Whereas, controls displayed a comparable number of omission errors across all three sessions, [t1 vs. t2: *t*_(17)_ = −1.29, *p* = 0.22, *ns*; t2 vs. t3: *t*_(17)_ = 1.37, *p* = 0.19, *ns*; t1 vs. t3: *t*_(17)_ = 0.20, *p* = 0.84, *ns*], children with ADHD improved in the second and third session when compared with the first session [t1 vs. t2: *t*_(15)_ = 3.03, *p* = 0.008; t2 vs. t3: *t*_(15)_ = −0.69, *p* = 0.50, *ns*; t1 vs. t3: *t*_(15)_ = 3.34, *p* = 0.005]. As a consequence, controls outperformed children with ADHD only at the first session, *F*_(1, 31)_ = 9.51, *p* = 0.004, η_*p*_^2^ = 0.24, but not at the second, *F*_(1, 31)_ = 2.68, *p* = 0.11, *ns*, or third session, *F*_(1, 31)_ = 0.16, *p* = 0.69, *ns*. With regard to the response time variability, controls again displayed a stable performance across all three sessions [t1 vs. t2: *t*_(17)_ = −1.06, *p* = 0.30, *ns*; t2 vs. t3: *t*_(17)_ = 0.25, *p* = 0.81, *ns*; t1 vs. t3: *t*_(17)_ = −0.73, *p* = 0.48, *ns*], whereas children with ADHD displayed increased response time variability during the third session when compared with the second session [t1 vs. t2: *t*_(15)_ = 1.69, *p* = 0.11, *ns*; t2 vs. t3: *t*_(16)_ = −3.01, *p* = 0.009; t1 vs. t3: *t*_(16)_ = −0.82, *p* = 0.43, *ns*]. As the group comparison shows, children with ADHD tended to display an increased response time variability at the first session, *F*_(1, 31)_ = 3.84, *p* = 0.06, η_*p*_^2^ = 0.11, and at the third session, *F*_(1, 31)_ = 3.06, *p* = 0.09, η_*p*_^2^ = 0.09, but not at the second session, *F*_(1, 31)_ = 1.32, *p* = 0.26, *ns*, when compared with controls.

**Figure 1:**
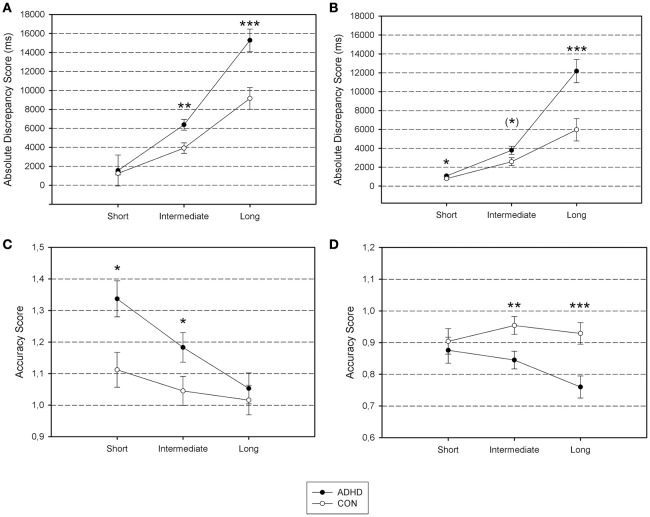
**Differential group effects of time interval length on performance accuracy. (A)** Time Production Absolute Error; **(B)** Time Reproduction Absolute Error; **(C)** Time Estimation Accuracy; **(D)** Time Reproduction Accuracy. Absolute Discrepancy Score, the absolute value of the deviation between specified and produced time interval in milliseconds (Time Production, Time Reproduction); Accuracy Score, the estimated time interval divided by the time interval to be produced in order to evaluate under- or overestimation in milliseconds (Time Estimation, Time Reproduction). Short, short time intervals (2, 6 s); Intermediate, intermediate time intervals (12, 24 s); Long, long time intervals (36, 48 s). ADHD, ADHD group; CON, control group. ^***^p < 0.001; ^**^p < 0.01; ^*^p < 0.05; ^(*)^*p* < 0.10.

**Figure 2:**
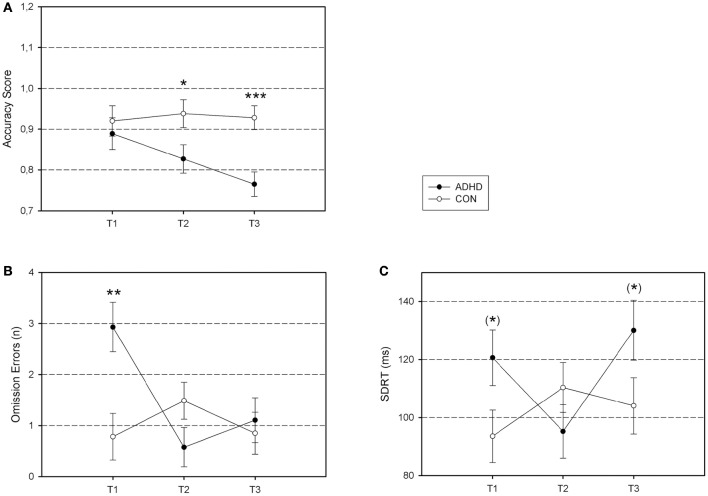
**Differential group effects of repeated task exposure. (A)** Time Reproduction Accuracy; **(B)** CPT Omission Errors; **(C)** CPT Response Time Variability. Accuracy Score, the estimated time interval divided by the time interval to be produced in order to evaluate under- or overestimation in milliseconds (Time Reproduction); Omission Errors, number of targets that were not identified (CPT); SDRT, standard deviation of reaction time for correctly identified targets in milliseconds (CPT). ADHD, ADHD group; CON, control group. ^***^p < 0.001; ^**^p < 0.01; ^*^p < 0.05; ^(*)^*p* < 0.10.

Significant within-between-subject interaction effects for the timing tasks (time intervals × diagnostic group) are presented in Figure 1. Significant within-between-subject interaction effects of repeated task completion (session × diagnostic group effects) are presented in Figure 2. All figures are based on the estimated marginal means.

### Cross-task correlations

Based on the data from the first session, analyses revealed significant correlations across all children between the time estimation and time production measures, whereas the time discrimination and time reproduction measures were not associated with any other task: The time estimation and time production accuracy were negatively correlated, *r* = −0.46, *p* = 0.006, whereas the absolute errors in both tasks were positively correlated, *r* = 0.54, *p* = 0.001. Correcting the global α-level for multiple comparisons by using the Bonferroni–Holm procedure (Holm, 1979), the association between the absolute errors remained significant (α_adj_ = 0.0024), whereas the association between the accuracy scores narrowly missed significance (global α_adj_ = 0.0025).

### Regression analyses

First, correlation analyses were conducted in order to examine associations between the potential predictor variables (*WM*: digit span, digit span backwards; *inattention*: number of omission errors, MDRT, and SDRT in the CPT; *impulsivity*: number of CPT commission errors, *ADHD symptom domains*: FBB-HKS subscale ratings for inattention, hyperactivity, and impulsivity), as well as age and IQ, with timing task performance (sensitivity threshold in the time discrimination task; absolute errors in the remaining tasks; see Table 3). Due to insignificant correlations with timing task performance, age, digit span backward, CPT commission errors, and MDRT were excluded as predictors. In a next step, intercorrelations between the predictor variables were analyzed and those with high intercorrelations but low association with the dependent variables were excluded in order to minimize problems with multicollinearity (Table 4). As the FBB-HKS ratings were highly intercorrelated and did not show dimensional specifity, they were excluded from further analyses. Likewise, digit span and digit span backwards were highly intercorrelated such that the digit span backwards was excluded as it showed only a marginal correlation with the time production error. Digit span, CPT omission errors, SDRT, and IQ remained as predictors for the subsequent regression analyses. Finally, simultaneous multiple regression analyses with the above specified predictors were performed. In order to assess the account of the digit span, CPT omission errors, and SDRT in explaining variation in the timing measures beyond the effect of differences in general intellectual ability, IQ was entered as a first predictor, whereas the remaining variables were simultaneously entered as predictors in a second step. We found that time discrimination threshold was predicted by IQ only, *F*_(1, 31)_ = 6.76, *p* = 0.01, $R_{\text{adjust}}^{2}$ = 0.15; β = −0.42, *t* = −2.60, *p* = 0.01, and the remaining variables did not predict variation in the time discrimination threshold beyond the account of IQ, [$R_{\text{change}}^{2}$, *F*_(3, 28)_ = 0.04, *p* = 0.71, *ns*]. Moreover, CPT omission errors solely predicted the time production absolute error, *F*_(4, 28)_ = 2.67, *p* = 0.05, $R_{\text{adjust}}^{2}$ = 0.17, β = 0.36, *t* = 2.09, *p* = 0.05, and the digit span solely predicted the time reproduction absolute error, *F*_(4, 28)_ = 3.68, *p* = 0.02, $R_{\text{adjust}}^{2}$ = 0.25, β = −0.36, *t* = −2.16, *p* = 0.04. Lastly, we explored if diagnostic group membership accounts for additional variation in the timing measures. For this reason, we implemented group in a third step in our regression analyses. For none of the models, significant changes in *R*^2^ emerged.

**Table 3:** **Correlations between predictor variables and timing task measures**.

	**Time discrimination threshold**	**Time estimation absolute error**	**Time production absolute error**	**Time reproduction absolute error**
Age	−0.01	−0.11	−0.06	−0.19
IQ	−0.43^*^	0.07	−0.24	−0.25
Inattention	0.09	0.18	0.56^**^	0.44^*^
Hyperactivity	−0.03	0.29	0.55^**^	0.33^*^
Impulsivity	−0.06	0.35^*^	0.65^**^	0.18
Digit span	0.01	−0.07	−0.21	−0.50^**^
Digit span backwards	0.11	−0.22	−0.29	−0.12
CPT omission errors	0.21	0.20	0.45^**^	0.39^*^
CPT commission errors	0.19	−0.09	−0.10	−0.02
MDRT	0.07	−0.17	0.07	0.12
SDRT	0.17	0.05	0.36^*^	0.31^(*)^

*Time Discrimination Threshold, the point at which two stimuli that are presented in the range of milliseconds and that differ in length are perceived as being equal (Time Discrimination); Absolute Error, the absolute value of the deviation between specified and produced time interval in milliseconds (Time Estimation, Time Production, Time Reproduction). Inattention, FBB-HKS inattention subscale; Hyperactivity, FBB-HKS hyperactivity subscale; Impulsivity, FBB-HKS impulsivity subscale. Digit Span, number of digits repeated; Digit Span Backwards, number of digits repeated backwards (Working Memory); CPT Omission Errors, number of targets that were not identified; CPT Commission Errors, non-targets that were identified as targets “A-not-X”); MDRT, median reaction time in milliseconds; SDRT, standard deviation of reaction time for correctly identified targets in milliseconds (CPT)*.

(*) p < 0.10;

* p < 0.05;

** *p < 0.01*.

**Table 4:** **Intercorrelations between predictor variables**.

	**Inattention**	**Hyper-activity**	**Impulsivity**	**Digit span**	**Digit span backwards**	**CPT omission errors**	**CPT commission errors**	**MDRT**	**SDRT**
Inattention									
Hyperactivity	0.81^***^								
Impulsivity	0.74^***^	0.87^***^							
Digit span	−0.24	−0.10	−0.11						
Digit span backwards	−0.03	−0.18	−0.14	0.46^**^					
CPT omission errors	0.50^**^	0.37^*^	0.50^**^	−0.23	−0.06				
CPT commission errors	−0.04	−0.14	−0.20	−0.02	−0.17	−0.02			
MDRT	−0.02	0.03	0.08	−0.32^(*)^	−0.21	0.05	−0.45^**^		
SDRT	0.16	0.10	0.17	−0.40^*^	−0.29^(*)^	0.23	−0.07	0.66^***^	

*Inattention, FBB-HKS inattention subscale; Hyperactivity, FBB-HKS hyperactivity subscale; Impulsivity, FBB-HKS impulsivity subscale. Digit Span, number of digits repeated; Digit Span Backwards, number of digits repeated backwards (Working Memory); CPT Omission Errors, number of targets that were not identified; CPT Commission Errors, non-targets that were identified as targets “A-not-X”); MDRT, median reaction time in milliseconds; SDRT, standard deviation of reaction time for correctly identified targets in milliseconds (CPT)*.

(*) p < 0.10;

* p < 0.05;

** p < 0.01;

*** *p < 0.001*.

## Discussion

In the present study, we comprehensively assessed perceptual timing task performance in boys with ADHD, using a broad range of tasks incorporating time discrimination, time estimation, time production, and time reproduction tasks. Moreover, we aimed to detect predictors responsible for the timing task performance deficits in children with ADHD and how these deficits might be positively affected by methylphenidate. Within our repeated measurements design, all timing tasks apart from the time reproduction task proved to be long-term stable measures of performance, i.e., no session-dependent performance differences emerged, making these tasks applicable for longitudinal studies.

Our first hypothesis was partly confirmed. As assumed, we found children with ADHD to perform worse than controls in the time discrimination and in the time reproduction task; however, deficits became also evident in the time estimation and in the time production tasks. More specifically, children with ADHD were less able than controls to discriminate short time intervals in the millisecond range, and in the range of several seconds, these children yielded larger absolute deviations from the time intervals to be estimated during the verbal estimation, as well as the manual production and reproduction, of these intervals. In the time production and in the time reproduction task, the error increased contingently on interval length in the ADHD group when compared with the control group. With regard to error direction, children with ADHD verbally overestimated the time intervals (i.e., they specified a higher numerical value in seconds), especially those intervals with short and intermediate durations, and they subsequently manually underproduced these time intervals (i.e., they indicated shorter periods of button presses). In the time reproduction task, children with ADHD manually underreproduced the time intervals, as well. Whereas, deficits in the time discrimination and time reproduction task fit well into the literature, time estimation and time production deficits were rather unexpected, but they might be attributable to shorter interval lengths (Meaux and Chelonis, 2003; Bauermeister et al., 2005) and to an older age of the subjects (Barkley et al., 2001) in previous research which might have masked these deficits. In line with the latter interpretation, age turned out as a significant covariate in our timing task analyses. Taken together, our results point to a general perceptual timing deficit in children with ADHD which seems not restricted to a specific subset of timing tasks.

Analyzing potential relationships between performances in the different timing tasks, we found significant associations between the time estimation and the time production task but a lack of correlation between the remaining tasks. This pattern suggests that independent timing task mechanisms may exist for discriminating, estimating/producing, and reproducing temporal intervals. Concerning the estimation/production mechanism, our results suggest that overestimation of temporal intervals is associated with underproduction of these intervals, resulting in increased absolute errors in both tasks, a finding which has already been described in non-ADHD samples (see Noreika et al., 2013, p. 238). Taking into consideration the association between time estimation and time production as a fundamental timing mechanism in humans as well as or results of interval overestimation and interval underproduction in children with ADHD which are in line with recent findings by others (Marx et al., 2010; Hurks and Hendriksen, 2011; Huang et al., 2012), one may assume an abnormally fast counting process as the underlying cause of timing dysfunctions in children with ADHD in these two tasks. As correlations have also been found between time estimation and time reproduction tasks in other studies (Bauermeister et al., 2005), although not consistently (Smith et al., 2002) or not adjusted for multiple comparisons (Hurks and Hendriksen, 2011), and interval underreproduction is prominent in children and adolescents with ADHD as well (Kerns et al., 2001; McInerney and Kerns, 2003), abnormally fast counting processes might be a prerequisite for time reproduction deficits in children with ADHD as well, although a lack of association between both tasks in our study suggests that different mechanisms might still be more important.

With regard to potential predictors of disturbed timing, we expected WM performance to predict time reproduction deficits in particular, and we expected measures of inattention and inhibition to predict timing dysfunctions as well, which should be especially true for neuropsychological measures when compared with observational measures in terms of parental behavioral symptom ratings. As expected, digit span as a measure of information storage and updating in WM (Owen et al., 2000; St. Clair-Thompson, 2010) by auditory rehearsal processes (Baddeley, 2003) negatively predicted the time reproduction absolute error, whereby poor performance accuracy in terms of interval underreproduction in the ADHD group supports the assumption of an abnormally fast counting process in these children. Comparing our results with previous findings, associations with time reproduction measures have been reported both for the storage and updating (Bauermeister et al., 2005) and for the manipulation (McInerney and Kerns, 2003) component of WM in children with ADHD. These findings might be explained by recent research demonstrating correlations between performance in the digit span and the digit span backwards task which is accompanied by neurofunctional overlap during tasks performance, showing that these functions are not completely functionally segregated from each other (Yang et al., 2015). Additionally, we found CPT omission errors as a measure of inattention (Allan and Lonigan, 2015) to positively predict the time production absolute error which was elevated in children with ADHD in our study. The dysfunctional mechanism here could be disruptions in counting by losing the train of thought, which has been clinically observed but not systematically evaluated in some of the children who participated in our study. However, we did not find WM to predict time discrimination deficits in ADHD. An in-depth analysis of task parameters from time discrimination studies that have been conducted so far reveals that WM was associated with time discrimination parameters when either stimulus length and/or inter-stimulus-interval were rather long (≥800 ms; Toplak et al., 2003; Toplak and Tannock, 2005; Yang et al., 2007) than short (Toplak and Tannock, 2005; Vloet et al., 2010), suggesting that WM is only crucial for timing performance from about 1 s and longer, when temporal characteristics of the experimental stimuli have to be analyzed and information has to be held in mind until the comparison stimulus is displayed. As we used both long stimulus baseline duration and a long inter-stimulus-interval, it seems most plausible that statistical power in our sample was too small to detect the assumed association. We found higher IQs to predict lower time discrimination thresholds, but the cognitive mechanism behind this umbrella term remains unclear. So far, no convincing evidence exists that time discrimination is impaired in children with ADHD when WM involvement is not required, a question that should be explored in future studies.

The ADHD symptom ratings were highly intercorrelated in our study and thus did not reflect specific ADHD sub-domains, but rather a general ADHD factor. Group-specific *post-hoc* analyses revealed that this correlation pattern was true in the ADHD group only where ceiling effects could additionally be observed, whereas these scales were uncorrelated in the control group. Therefore, we argue that ADHD rating scales, at least when the information is received from the parents, might not be suitable in predicting timing deficits in ADHD samples, giving point to neuropsychological measures of these dimensions as the more appropriate predictors.

In contrast to our expectations, we did not find MPH to improve timing task performance in children with ADHD. Differential group effects of repeated task exposure emerged for the time reproduction task and for the CPT only. In the time reproduction task, only children with ADHD, but not controls, underreproduced the time intervals with increasing number of task repetitions, i.e., their performance accuracy decreased in the course of the experimental sessions. As a result, children with ADHD displayed significantly decreased intra individual performance accuracy and marked group differences emerged during the third experimental session. Notably, this performance pattern was specific to the time reproduction task, whereas performance in the remaining timing tasks was stable. This finding suggests that mechanisms different from MPH medication (which would have produced superior performance during the second session when compared with the first and the third session) and different from effects of practice (which would have produced increasing performance accuracy with increasing session number) came into operation in the ADHD sample during repeated time reproduction task exposure. As this task constitutes the longest of the timing tasks with the highest demands on patience, these mechanisms might be motivational by nature. In this sense, delay aversion, i.e., the motivational tendency to avoid delays over time as they are perceived as emotionally aversive (Sonuga-Barke, 2003), might turn out to be a promising construct in explaining time reproduction deficits in ADHD, but research on this issue is lacking so far. Indeed, reward related to performance was found to reduce (McInerney and Kerns, 2003) or even to disperse (Marx et al., 2013) time reproduction deficits in ADHD in earlier studies, arguing for motivational involvement in time reproduction task performance.

A divergent response pattern of repeated task exposure was observed for the CPT: Children with ADHD displayed a decrease of omission errors in the second session when compared with the first session, whereas controls did not. Moreover, children with ADHD displayed a trend for increased response time variability during the first and the third session when compared with the second session. Whereas the former effect might be interpreted as an effect of practice (as the number of omission errors does not re-deteriorate during the third session) the latter one might be interpreted cautiously as a MPH effect (as performance tends to re-deteriorate during the third session after tendential improvement in the second session, where the children were medicated with MPH). Thus, although MPH might have slightly improved attention as indicated by decreased CPT response time variability and in accordance with recent meta-analytical evidence (Kofler et al., 2013), timing functions were not affected in our ADHD sample. However, this interpretation must be treated with caution, as our study was limited by a small sample size and we might therefore have failed to detect significant MPH effects. Indeed, two recently conducted studies found MPH to reduce time discrimination deficits in children and adolescents with ADHD (Smith et al., 2013; Rubia et al., 2014). As a number of further studies also failed to detect drug effects, probably due to limited statistical power on the basis of small sample sizes (Barkley et al., 1997; Rubia et al., 2003, 2009), our knowledge about MPH effects on perceptual timing task performance in children with ADHD is still limited, and it is almost exclusively restricted on the time discrimination paradigm. As a consequence, there is an urgent need for replication studies across all four perceptual timing domains using larger sample sizes in order to assess possible MPH effects independent of power limitations.

Summarizing the results of our study, we found that overestimation of temporal intervals (i.e., reporting higher numerical estimates in seconds with regard to the actually presented time interval) was associated with underproduction of these intervals (i.e., producing shorter time intervals than actually indicated) in all children, although this finding narrowly missed significance after adjusting for multiple comparisons. In addition, we found that children with ADHD both verbally overestimated the time intervals in the time estimation task and manually underproduced these intervals in the time production task when compared with controls. These findings argue for an abnormally fast counting process in children with ADHD. Putatively contradicting this assumption, the time production error in our sample was not predicted by WM measures, but by CPT omission errors. However, as CPT omission errors were negatively correlated with the digit span, *r* = −0.50, *p* = 0.05, but not with digit span backwards, *r* = −0.22, *p* = 0.41, *ns*, it stands to reason that CPT omission errors might be understood, at least in part, as being associated with deficits in holding and updating information within the WM. Therefore, we put disturbed WM processes in terms of an abnormally fast counting process as well as attention problems (e.g., in terms of disruptions in counting) as the reasons for time estimation and time production deficits in children with ADHD forward for discussion. Furthermore, time reproduction deficits were predicted by the digit span, bringing disturbed rehearsal processes within the WM for this task up for discussion, as well. In doing so, underreproduction of the specified time intervals in children with ADHD also argues for an abnormally fast counting process during task performance. Moreover, the interaction effect between time reproduction error and session number suggests an additional impact of motivational factors on task performance, turning out an abnormally fast counting process as well as motivational deficits (e.g., in terms of increased delay aversion) as potential reasons for time reproduction deficits in children with ADHD.

### Conclusions

Children with ADHD seem to suffer from a general perceptual timing deficit which is not restricted to specific timing tasks. In doing so, we suggest three distinct but partially overlapping neurocognitive mechanisms for discriminating, estimating/producing, and reproducing temporal intervals which seem impaired in these children: WM deficits might be common to timing dysfunctions in time estimation/time production tasks and in time reproduction tasks, with attention deficits additionally contributing to time estimation/time production deficits and motivational alterations additionally contributing to time reproduction deficits. These findings support the Barkley (1997) model in that WM seems crucial for timing abilities, independent from the underlying paradigms and at least for tasks in the seconds range. However, very first evidence from our longitudinal study tentatively pointing to motivational aspects of impaired time reproduction performance suggests that at least a part of the timing deficits in children with ADHD might be better explained within the framework of the dual pathway model of ADHD (Sonuga-Barke, 2003) that specifies both executive and motivational pathways in explaining symptoms of the disorder. With regard to time discrimination performance, the question is unsolved so far if children with ADHD are impaired at all when WM is not required.

Given the central role of WM in the performance across different perceptual timing tasks as suggested by our results, it is an issue of ongoing debate if WM reflects an intrinsic part of a central clock mechanism which is disturbed in ADHD as represented by abnormally fast counting processes (i.e., a faster “internal clock”) or if WM—together with further executive support functions—and perceptual timing share common neurofunctional networks such that timing performance in children with ADHD is confounded by executive dysfunctions (Rubia et al., 2009; Noreika et al., 2013). As successive underreproduction with increasing interval length is well replicated, at least for the time reproduction task, and as we found decreasing performance accuracy with increasing session number in this task within our longitudinal study design, we would like to broaden the current debate by making a third suggestion that timing performance in children with ADHD might be confounded by motivational deficits resulting from increased delay aversion. In other words, it is conceivable that WM dysfunctions in terms of faster internal counting processes in children with ADHD might mediate the relationship between delay aversion and temporal under(re)production in perceptual timing tasks. As delay aversion increases, by definition, with time on task, it should manifest itself by an increasingly faster counting process with time on task, whereas the counting process should be already initially faster if internal clock/executive dysfunctions predominate. In order to further specify the mechanisms contributing to impaired perceptual timing in children with ADHD, future studies should implement loud counting strategies in order to enable speed analyses and the detection of disruptions in the counting sequence, and they should implement measures that capture motivational and emotional aspects during time on task.

## Author contributions

IM: conception and design of the study, acquisition, analysis, and interpretation of data, drafting the manuscript, and final approval. SW: conception of the study, acquisition of data, revising the manuscript critically for important intellectual content, and final approval. CB: analysis and interpretation of data, revising the manuscript critically for important intellectual content, and final approval. SH: conception of the study, revising the manuscript critically for important intellectual content, and final approval. SC and JH: interpretation of data, revising the manuscript critically for important intellectual content, and final approval. RW and FH: conception and design of the study, revising the manuscript critically for important intellectual content and final approval Agreement to be accountable for all aspects of the work in ensuring that questions related to the accuracy or integrity of any part of the work are appropriately investigated and resolved is stated by all authors.

### Conflict of interest statement

The authors declare that the research was conducted in the absence of any commercial or financial relationships that could be construed as a potential conflict of interest.

## References

[B1] Allan D. M. Lonigan C. J. (2015). Relations between response trajectories on the continuous performance test and teacher-rated problem behaviors in preschoolers. Psychol. Assess. 27, 678–688. 10.1037/pas0000054 25419645 PMC4442070

[B2] American Psychiatric Association (2013). Diagnostic and Statistical Manual of Mental Disorders, 5th Edn. Arlington, TX: American Psychiatric Association.

[B3] Baddeley A. (1992). Working memory. Science 255, 556–559. 10.1126/science.1736359 1736359

[B4] Baddeley A. (2003). Working memory: looking back and looking forward. Nat. Rev. Neurosci. 4, 829–839. 10.1038/nrn1201 14523382

[B5] Barkley R. A. (1997). Attention-deficit/hyperactivity disorder, self-regulation, and time: toward a more comprehensive theory. J. Dev. Behav. Pediatr. 18, 271–279. 10.1097/00004703-199708000-00009 9276836

[B6] Barkley R. A. Edwards G. Laneri M. Fletcher K. Metevia L. (2001). Executive functioning, temporal discounting, and sense of time in adolescents with attention deficit hyperactivity disorder (ADHD) and oppositional defiant disorder (ODD). J. Abnorm. Child Psychol. 29, 541–556. 10.1023/A:1012233310098 11761287

[B7] Barkley R. A. Koplowitz S. Anderson T. McMurray M. B. (1997). Sense of time in children with ADHD: effects of duration, distraction, and stimulant medication. J. Int. Neuropsychol. Soc. 3, 359–369. 9260445

[B8] Bauermeister J. J. Barkley R. A. Martinez J. V. Cumba E. Ramirez R. R. Reina G.. (2005). Time estimation and performance on reproduction tasks in subtypes of children with attention deficit hyperactivity disorder. J. Clin. Child Adolesc. Psychol. 34, 151–162. 10.1207/s15374424jccp3401_14 15677289

[B9] Challman T. D. Lipsky J. J. (2000). Methylphenidate: its pharmacology and uses. Mayo Clin. Proc. 75, 711–721. 10.1016/S0025-6196(11)64618-1 10907387

[B10] Coghill D. Banaschewski T. Zuddas A. Pelaz A. Gagliano A. Doepfner M. (2013). Long-acting methylphenidate formulations in the treatment of attention-deficit/hyperactivity disorder: a systematic review of head-to-head studies. BMC Psychiatry 13:237. 10.1186/1471-244X-13-237 24074240 PMC3852277

[B11] Coghill D. R. Seth S. Pedroso U. Usala T. Currie J. Gagliano A. (2014). Effects of methylphenidate on cognitive functions in children and adolescents with attention-deficit/hyperactivity disorder: evidence from a systematic review and a meta-analysis. Biol. Psychiatry 76, 603–615. 10.1016/j.biopsych.2013.10.005 24231201

[B12] Döpfner M. Lehmkuhl G. (1998). Diagnostic System for Mental Disorders in Child and Adolescence According to ICD-10 and DSM-IV (DISYPS-KJ). Bern: Hans Huber.

[B13] Grondin S. (2010). Timing and time perception: a review of recent behavioral and neuroscience findings and theoretical directions. Atten. Percept. Psychophys. 72, 561–582. 10.3758/APP.72.3.561 20348562

[B14] Härting C. Markowitsch H. J. Neufeld H. Calabrese P. Deisinger K. Kessler J. (2000). WMS-R Wechsler Gedächtnistest – Revidierte Fassung. Deutsche Adaptation der Revidierten Fassung der Wechsler Memory Scale. Manual. Bern: Hans Huber.

[B15] Himpel S. Banaschewski T. Grüttner A. Becker A. Heise A. Uebel H.. (2009). Duration discrimination in the range of milliseconds and seconds in children with ADHD and their unaffected siblings. Psychol. Med. 39, 1745–1751. 10.1017/S003329170900542X 19265568

[B16] Holm S. (1979). A simple sequentially rejective multiple test procedure. Scand. J. Stat. 6, 65–70.

[B17] Huang J. Bin-rang Y. Xiao-bing Z. Jin J. Gang P. Gr1inne M.. (2012). Temporal processing impairment in children with attention-deficit-hyperactivity disorder. Res. Dev. Disabil. 33, 538–548. 10.1016/j.ridd.2011.10.021 22119703

[B18] Hurks P. P. Hendriksen J. G. (2011). Retrospective and prospective time deficits in childhood ADHD: the effects of task modality, duration, and symptom dimensions. Child Neuropsychol. 17, 34–50. 10.1080/09297049.2010.514403 20936546

[B19] Hwang S. L. Gau S. S. Hsu W. Y. Wu Y. Y. (2010). Deficits in interval timing measured by the dual-task paradigm among children and adolescents with attention-deficit/hyperactivity disorder. J. Child Psychol. Psychiatry 51, 223–232. 10.1111/j.1469-7610.2009.02163.x 19754502

[B20] Kaufman J. Birmaher B. Brent D. Rao U. Flynn C. Moreci P.. (1997). Schedule for affective disorders and Schizophrenia for school-age children-present and lifetime version (K-SADSPL): initial reliability and validity data. J. Am. Acad. Child Adolesc. Psychiatry 36, 980–988. 10.1097/00004583-199707000-00021 9204677

[B21] Kerns K. A. McInerney R. J. Wilde N. J. (2001). Time reproduction, working memory, and behavioral inhibition in children with ADHD. Child Neuropsychol. 7, 21–31. 10.1076/chin.7.1.21.3149 11815878

[B22] Kimko H. C. Cross J. T. Abernethy D. R. (1999). Pharmacokinetics and clinical effectiveness of methylphenidate. Clin. Pharmacokinet. 37, 457–470. 10.2165/00003088-199937060-00002 10628897

[B23] Kofler M. J. Rapport M. D. Sarver D. E. Raiker J. S. Orban S. A. Friedman L. M.. (2013). Reaction time variability in ADHD: a meta-analytic review of 319 studies. Clin. Psychol. Rev. 33, 795–811. 10.1016/j.cpr.2013.06.001 23872284

[B24] Levitt H. (1971). Transformed up-down methods in psychoacoustics. J. Acoust. Soc. Am. 49, 467–477. 10.1121/1.1912375 5541744

[B25] Luman M. van Noesel S. J. Papanikolau A. Van Oostenbruggen-Scheffer J. Veugelers D. Sergeant J. A.. (2009). Inhibition, reinforcement sensitivity and temporal information processing in ADHD and ADHD+ODD: evidence of a separate entity? J. Abnorm. Child Psychol. 37, 1123–1135. 10.1007/s10802-009-9334-0 19543967 PMC2766046

[B26] Martinussen R. Hayden J. Hogg-Johnson S. Tannock R. (2005). A meta-analysis of working memory impairments in children with attention-deficit/hyperactivity disorder. J. Am. Acad. Child Adolesc. Psychiatry 44, 377–384. 10.1097/01.chi.0000153228.72591.73 15782085

[B27] Marx I. Höpcke C. Berger C. Wandschneider R. Herpertz S. C. (2013). The impact of financial reward contingencies on cognitive function profiles in adult ADHD. PLoS ONE 8:e67002. 10.1371/journal.pone.0067002 23840573 PMC3688618

[B28] Marx I. Hübner T. Herpertz S. C. Berger C. Reuter E. Kircher T.. (2010). Cross-sectional evaluation of cognitive functioning in children, adolescents and young adults with ADHD. J. Neural. Transm. 117, 403–419. 10.1007/s00702-009-0345-3 19953279

[B29] McInerney R. J. Kerns K. A. (2003). Time reproduction in children with ADHD: motivation matters. Child Neuropsychol. 9, 91–108. 10.1076/chin.9.2.91.14506 12815512

[B30] Meaux J. B. Chelonis J. J. (2003). Time perception differences in children with and without ADHD. J. Pediatr. Health Care 17, 64–71. 10.1067/mph.2003.26 12665728

[B31] Noreika V. Falter C. M. Rubia K. (2013). Timing deficits in attention-deficit/hyperactivity disorder (ADHD): evidence from neurocognitive and neuroimaging studies. Neuropsychologia 51, 235–266. 10.1016/j.neuropsychologia.2012.09.036 23022430

[B32] Owen A. M. Lee A. C. H. Williams E. J. (2000). Dissociating aspects of verbal working memory within the human frontal lobe: further evidence for a “process-specific” model of lateral frontal organization. Psychobiology 28, 146–155. 10.3758/BF03331974

[B33] Owen A. M. McMillan K. M. Laird A. R. Bullmore E. (2005). N-back working memory paradigm: a meta-analysis of normative functional neuroimaging studies. Hum. Brain Mapp. 25, 46–59. 10.1002/hbm.20131 15846822 PMC6871745

[B34] Patrick K. S. Markowitz J. S. (1997). Pharmacology of methylphenidate, amphetamine enantiomers and pemoline in attention-deficit hyperactivity disorder. Hum. Psychopharmacol. 12, 527–546. 10.1002/(SICI)1099-1077(199711/12)12:6<527::AID-HUP932>3.0.CO;2-U

[B35] Plummer C. Humphrey N. (2009). Time perception in children with ADHD: the effects of task modality and duration. Child Neuropsychol. 15, 147–162. 10.1080/09297040802403690 18825522

[B36] Prasad V. Brogan E. Mulvaney C. Grainge M. Stanton W. Sayal K. (2013). How effective are drug treatments for children with ADHD at improving on-task behaviour and academic achievement in the school classroom? A systematic review and meta-analysis. Eur. Child Adolesc. Psychiatry 22, 203–216. 10.1007/s00787-012-0346-x 23179416

[B37] Punja S. Zorzela L. Hartling L. Urichuk L. Vohra S. (2013). Long-acting versus short-acting methylphenidate for paediatric ADHD: a systematic review and meta-analysis of comparative efficacy. BMJ Open 3:e002312. 10.1136/bmjopen-2012-002312 23503579 PMC3612754

[B38] Rommelse N. N. Altink M. E. Oosterlaan J. Beem L. Buschgens C. J. Buitelaar J.. (2008). Speed, variability, and timing of motor output in ADHD: which measures are useful for endophenotypic research? Behav. Genet. 38, 121–132. 10.1007/s10519-007-9186-8 18071893 PMC2257997

[B39] Rommelse N. N. Oosterlaan J. Buitelaar J. Faraone S. V. Sergeant J. A. (2007). Time reproduction in children with ADHD and their nonaffected siblings. J. Am. Acad. Child Adolesc. Psychiatry 46, 582–590. 10.1097/CHI.0b013e3180335af7 17450049

[B40] Rottschy C. Langner R. Dogan I. Reetz K. Laird A. R. Schulz J. R.. (2012). Modelling neural correlates of working memory: a coordinate-based meta-analysis. Neuroimage 60, 830–846. 10.1016/j.neuroimage.2011.11.050 22178808 PMC3288533

[B41] Rubia K. Alegria A. A. Cubillo A. I. Smith A. B. Brammer M. J. Radua J. (2014). Effects of stimulants on brain function in attention-deficit/hyperactivity disorder: a systematic review and meta-analysis. Biol. Psychiatry 76, 616–628. 10.1016/j.biopsych.2013.10.016 24314347 PMC4183380

[B42] Rubia K. Halari R. Christakou A. Taylor E. (2009). Impulsiveness as a timing disturbance: neurocognitive abnormalities in attention-deficit hyperactivity disorder during temporal processes and normalization with methylphenidate. Philos. Trans. R. Soc. B 364, 1919–1931. 10.1098/rstb.2009.0014 19487194 PMC2685816

[B43] Rubia K. Noorloos J. Smith A. Gunning B. Sergeant J. (2003). Motor timing deficits in community and clinical boys with hyperactive behavior: the effect of methylphenidate on motor timing. J. Abnorm. Child Psychol. 31, 301–313. 10.1023/A:1023233630774 12774863

[B44] Rubia K. Smith A. Taylor E. (2007). Performance of children with attention deficit hyperactivity disorder (ADHD) on a test battery of impulsiveness. Child Neuropsychol. 13, 276–304. 10.1080/09297040600770761 17453834

[B45] Smith A. B. Taylor E. Brammer M. Halari R. Rubia K. (2008). Reduced activation in right lateral prefrontal cortex and anterior cingulate gyrus in medication-naïve adolescents with attention deficit hyperactivity disorder during time discrimination. J. Child Psychol. Psychiatry 49, 977–985. 10.1111/j.1469-7610.2008.01870.x 18759938

[B46] Smith A. Cubillo A. Barrett N. Giampietro V. Simmons A. Brammer M.. (2013). Neurofunctional effects of methylphenidate and atomoxetine in boys with attention-deficit/hyperactivity disorder during time discrimination. Biol. Psychiatry 74, 615–622. 10.1016/j.biopsych.2013.03.030 23731741

[B47] Smith A. Taylor E. Rogers J. W. Newman S. Rubia K. (2002). Evidence for a pure time perception deficit in children with ADHD. J. Child Psychol. Psychiatry 43, 529–542. 10.1111/1469-7610.00043 12030598

[B48] Sonuga-Barke E. J. S. (2003). The dual pathway model of AD/HD: an elaboration of neuro-developmental characteristics. Neurosci. Biobehav. Rev. 27, 593–604. 10.1016/j.neubiorev.2003.08.005 14624804

[B49] St. Clair-Thompson H. L. (2010). Backwards digit recall: a measure of short-term memory or working memory? Eur. J. Cogn. Psychol. 22, 86–296. 10.1080/09541440902771299

[B50] Toplak M. E. Rucklidge J. J. Hetherington R. John S. C. Tannock R. (2003). Time perception deficits in attention-deficit/ hyperactivity disorder and comorbid reading difficulties in child and adolescent samples. J. Child Psychol. Psychiatry 44, 888–903. 10.1111/1469-7610.00173 12959497

[B51] Toplak M. E. Tannock R. (2005). Time perception: modality and duration effects in attention-deficit/hyperactivity disorder (ADHD). J. Abnorm. Child Psychol. 33, 639–654. 10.1007/s10802-005-6743-6 16195956

[B52] Valko L. Schneider G. Doehnert M. Müller U. Brandeis D. Steinhausen H. C.. (2010). Time processing in children and adults with ADHD. J. Neural Transm. 117, 1213–1228. 10.1007/s00702-010-0473-9 20821338

[B53] Van Meel C. S. Oosterlaan J. Heslenfeld D. J. Sergeant J. A. (2005). Motivational effects on motor timing in attention-deficit/hyperactivity disorder. J. Am. Acad. Child Adolesc. Psychiatry 44, 451–460. 10.1097/01.chi.0000155326.22394.e6 15843767

[B54] Vloet T. D. Gilsbach S. Neufang S. Fink G. R. Herpertz-Dahlmann B. Konrad K. (2010). Neural mechanisms of interference control and time discrimination in attention-deficit/hyperactivity disorder. J. Am. Acad. Child Adolesc. Psychiatry 49, 356–367. 10.1097/00004583-201004000-00010 20410728

[B55] Wager T. D. Smith E. E. (2003). Neuroimaging studies of working memory: a meta-analysis. Cogn. Affect. Behav. Neurosci. 3, 255–274. 10.3758/CABN.3.4.255 15040547

[B56] Weiss R. H. (1998). Grundintelligenztest Skala 2 (CFT 20). Gottingen: Hogrefe.

[B57] Weiss R. H. Osterland J. (1997). Grundintelligenztest Skala 1 (CFT1). Gottingen: Hogrefe.

[B58] Wiener M. Turkeltaub P. Coslett H. B. (2010). The image of time: a voxel-wise meta-analysis. Neuroimage 49, 1728–1740. 10.1016/j.neuroimage.2009.09.064 19800975

[B59] Yang B. Chan R. C. Zou X. Jing J. Mai J. Li J. (2007). Time perception deficit in children with ADHD. Brain Res. 1170, 90–96. 10.1016/j.brainres.2007.07.021 17669375

[B60] Yang Z. Jutagir D. R. Koyama M. S. Craddock R. C. Yan C. G. Shehzad Z.. (2015). Intrinsic brain indices of verbal working memory capacity in children and adolescents. Dev. Cogn. Neurosci. 15, 67–82. 10.1016/j.dcn.2015.07.007 26299314 PMC4696540

